# Нормофосфатемический семейный опухолевый кальциноз: описание клинического случая с назначением гипофосфатемической терапии

**DOI:** 10.14341/probl13713

**Published:** 2026-09-08

**Authors:** А. В. Витебская, Ю. В. Тихонович, А. А. Колодкина

**Affiliations:** Первый МГМУ им. И.М. Сеченова Минздрава России (Сеченовский Университет) Россия; Sechenov First Moscow State Medical University Russian Federation; Первый МГМУ им. И.М. Сеченова Минздрава России (Сеченовский Университет); Российский национальный исследовательский медицинский университет им. Н.И. Пирогова; Медико-генетический научный центр им. академика Н.П. Бочкова Россия; Sechenov First Moscow State Medical University; Russian National Research Medical University named after N.I. Pirogov; Medical Genetic Research Center named after Academician N.P. Bochkov Russian Federation; Национальный медицинский исследовательский центр эндокринологии им. академика И.И. Дедова Россия; Endocrinology Research Center Russian Federation

**Keywords:** нормофосфатемический семейный опухолевый кальциноз, кальциноз кожи, SAMD9, севеламер, normophosphatemic familial tumoral calcinosis, calcinosis cutis, SAMD9, sevelamer

## Abstract

Нормофосфатемический семейный опухолевый кальциноз (НСОК) характеризуется наличием кожных кальцинатов при отсутствии метаболических нарушений; манифестирует в первые годы жизни; ассоциирован с мутациями в гене SAMD9. В образовании кальцинатов ведущую роль играют хроническая травматизация и транзиторная гиперфосфатемия.

Хирургическое лечение позволяет избавляться от кальцинатов, но не предотвращает рецидивы и может приводить к осложнениям. Применяются различные методы консервативной терапии. Снижение уровня фосфатов в крови рассматривается как перспективный патогенетический метод лечения.

В статье представлен клинический случай пациента с НСОК, ассоциированный с гомозиготным вариантом p.E1200K в SAMD9. Особенностью нашего пациента является наличие множественных кальцинатов не только в местах травматизации (конечности), но и в местах угревой сыпи (лицо), а также особенно активное прогрессирование заболевания в период полового созревания. Уменьшение размеров кальцинатов отмечено на фоне 1 года постоянной гипофосфатемической терапии (гидроксид алюминия и севеламер 800 мг). Эффективность проводимой терапии дискутабельна, ограничена как плохой переносимостью севеламера пациентом, так и недостаточной приверженностью к лечению.

Это первое в России описание пациента с НСОК, подтвержденным молекулярно-генетически. Точная постановка диагноза позволила уточнить патогенетический механизм образования кальцинатов и предложить варианты терапии.

## Abstract (en)

Normophosphatemic familial tumoral calcinosis (NFTC) is characterized by cutaneous calcifications with no metabolic disorders; it manifests in the first years of life; associated with mutations in SAMD9 gene. Chronic trauma and transient hyperphosphatemia play a leading role in calcification formation.

Surgical treatment can remove calcifications, but it does not prevent recurrence and can cause complications. Various conservative therapy is used. Lowering of blood phosphate levels is considered a promising pathogenetic treatment method.

We present a clinical case of a patient with NFTC associated with the homozygous variant p.E1200K in SAMD9. A characteristic feature of our patient is the presence of multiple calcifications not only in the areas of injury (limbs), but also in areas of acne (face), as well as particularly active progression of the disease during puberty. A reduction in calcification size was observed after one year of continuous hypophosphatemic therapy (aluminum hydroxide and sevelamer 800 mg). The effectiveness of this therapy is debatable and limited by both poor patient tolerability of sevelamer and poor adherence.

This is the first description of a patient with molecularly confirmed NFTC in Russia. A precise diagnosis allowed us to clarify the pathogenetic mechanism of calcification formation and propose treatment options.

## АКТУАЛЬНОСТЬ

Эндокринологи нередко сталкиваются с отложением солей кальция в различных органах и тканях у пациентов с нарушениями кальций-фосфорного обмена. Кальцинаты кожи могут также формироваться на фоне хронических системных воспалительных заболеваний, а также при ряде редких генетических патологий, приводящих к гетеротопической оссификации [1].

Такие пациенты, как правило, направляются к эндокринологу для выявления возможных нарушений кальций-фосфорного обмена и подбора патогенетической терапии. Однако ввиду редкой встречаемости и недостаточной освещенности подобных заболеваний в литературе процесс диагностики может занимать длительное время. Во многих случаях точная нозологическая верификация невозможна без применения молекулярно-генетических методов исследования.

## ОПИСАНИЕ СЛУЧАЯ

## Анамнез жизни

Мальчик от физиологически протекавшей беременности, срочных родов путем кесарева сечения из-за слабости родовой деятельности. При рождении: вес — 3200 г, длина — 51 см, закричал сразу. В возрасте 3 мес в медицинской документации зафиксирована гиперэргическая реакция на АКДС, потребовавшая госпитализации. В 1 год отмечена аллергическая реакция на молоко; при проведении обследования выявлена поливалентная аллергия.

Рост родителей: 178 см (папа) и 165 см (мама). Наследственность отягощена по сахарному диабету 2 типа и мочекаменной болезни. У родителей пациента нарушения кальций-фосфорного обмена и костные деформации не выявлены.

## Анамнез заболевания

В возрасте 1 года впервые обратили внимание на изменения кожи на кончиках пальцев рук после травмы, при обследовании диагностированы вульгарные бородавки. В 1 год 3 мес на правой кисти выявлено образование в области первого пястно-фалангового сустава, проведено удаление фибромы, содержавшей кальцинат.

В 4 года 7 мес пациент обследован в НИИ ревматологии по поводу формирующихся кожных кальцинатов. Данных за ревматическую патологию не получено; при эхокардиографии заподозрено наличие кальцината на некоронарной створке аортального клапана. Биопсия одного из подкожных образований подтвердила инкапсулированное отложение солей кальция.

В 4 года 10 мес впервые госпитализирован в детское эндокринологическое отделение Университетской детской клинической больницы. При осмотре массо-ростовые показатели соответствовали возрасту, телосложение пропорциональное (табл. 1). Значимых костных деформаций не выявлено: форма черепа округлая, роднички и швы закрыты, прогнатия; грудная клетка с незначительной воронкообразной деформацией; конечности пропорциональны, не искривлены, сандалевидная щель между 1 и 2 пальцами ног; позвоночник без патологических искривлений, ригидности нет, безболезненный при пальпации; суставы — форма не изменена, артралгий, ограничений движения нет. Обращали на себя внимание кальцинаты размером до 1 см в диаметре в области локтей, коленей, пальцев ног, на крыле носа справа, бородавки на пальцах ног и рук (табл. 1) (рис. 1А).

**Table:** Таблица 1. Клинико-лабораторные показатели пациента за весь период наблюдения Примечания: ¹ — неравномерное повышение плотности клапана легочной артерии не позволяет исключить наличие кальцината; ² — колебания фосфора на фоне подбора дозы севеламера; ³ — указаны рекомендованные дозы, которые пациент принимал нерегулярно.

Возраст	4 г. 10 мес	5 лет 11 мес	7 лет 1 мес	8 лет 1 мес	9 лет 1 мес	10 лет 1 мес	11 лет 2 мес	12 лет 5 мес	13 лет 3 мес	14 лет 3 мес	15 лет 4 мес	16 лет	17 лет
Рост, см (SDS)	104,6(-0,62)	110,3(-0,65)	117,5(-0,65)	121,0(-1,01)	126,7(-0,90)	131,0(-1,00)	132,8(-1,50)	138,5(-1,50)	143,5(-1,46)	155,0(-0,82)	163,4(-0,77)	165,0(-1,08)	167(-1,10)
Вес, кг, (SDS ИМТ)	15,7(-1,08)	17,4(-1,04)	19,5(-1,16)	21,1(-0,98)	22,9(-1,26)	25,1(-1,14)	24,9(-1,91)	29,5(-1,32)	31,3(-1,77)	38,0(-1,66)	47,4(-0,88)	49,0(-0,97)	50,4(-1,21)
Кальцинаты	Осмотр	нос	+	+	+	+	+	+	+	+	+	+	+	+	
лицо, шея			+	+	+	+	+	+	+	+	+	+	+
локти	+	+	+	+	+	+	+	+	+	+	+	+	+
кисти		+	+	+	+	+	+	+	+	+	+	+	+
ягодицы							+	+	+	+	+	+	+
колени	+	+	+	+	+	+	+	+	+	+	+	+	+
стопы	+	+	+	+	+	+	+	+	+	+	+	+	+
УЗИ	почки			2*3 мм	2*3 мм	2*2 мм								
мошонка				3*3 мм	7*4 мм	7*4 мм	7*7 мм	7*5 мм;1*2 мм	6*5 мм;2*2 мм	6*2 мм;2*3 мм	9*4 мм	4*3 мм	3,5*2 мм;3,9*1,6 мм
сердце											+(?)²	+(?)²	+(?)²
Кальций ионизир., ммоль/л (N 1,03–1,35)	1,11	1,38	1,23	1,31	1,22	1,23	1,35	1,3	1,26	1,34	1,17	1,25	1,23
Фосфор неорг., ммоль/л(норма по возрасту)	1,80(1,38–2,19)	1,60(1,33–1,92)	1,77(1,33–1,92)	-	-	1,57(1,33–1,92)	1,60(1,33–1,92)	1,7(1,33–1,92)	1,86(1,14–1,99)	2,02(1,14–1,99)	1,20–2,13²(1,14–1,99)	1,62–1,82²(0,95–1,62)	1,37–1,46–1,66(0,95–1,62)
Щелочная фосфатаза, Ед/л(норма по возрасту)	287(156–369)	-	548(156–369)	-	527(156–369)	627(141–460)	435(141–460)	657(141–560)	646(141–560)	300(141–560)	238(89–365)	195(89–365)	145(89–365)
Паратгормон, пмоль/л (N 1,3–10)	2,0	1,4	2,1	0,8	-	0,9	6,6	2,8	3,0	5,3	3,5	1,8	1,97
25-ОН-витамин Д, нг/мл (N>30)	28,9	-	-	-	8,8	-	-	-	18	24	33	42	51,8
Кальций/креатинин в моче, моль/ммоль(норма по возрасту)	0,15(0,05–1,1)	0,13(0,04–0,8)	0,24(0,04–0,8)	0,12(0,04–0,7)	0,18(0,04–0,7)	0,34(0,04–0,6)	0,18(0,04–0,6)	-	-	-	-	0,09(0,04–0,6)	-
Фосфор/креатинин в моче, моль/ммоль(норма по возрасту)	2,5(1,2–8,0)	4,2(1,2–5,0)	4,0(1,2–3,6)	4,2(1,2–3,6)	3,2(1,2–3,6)	3,82(0,8–3,2)	3,34(0,8–3,2)	-	-	-	-	1,82(0,8–2,7)	-
Предшествующая терапия	Витамин Д					1000 МЕ курсами, непостоянно	1000 МЕ³	1000 МЕ³	2500 МЕ	3000 МЕ	3000 МЕ
Магния гидроксид 600 мг, Алюминия гидроксид 525 мг											1 саше 2 раза в сутки³		1 саше 2 раза в сутки
Севеламер												800 мг 3 раза в сутки³	2 таб по 800 мг вечером

**Figure:**
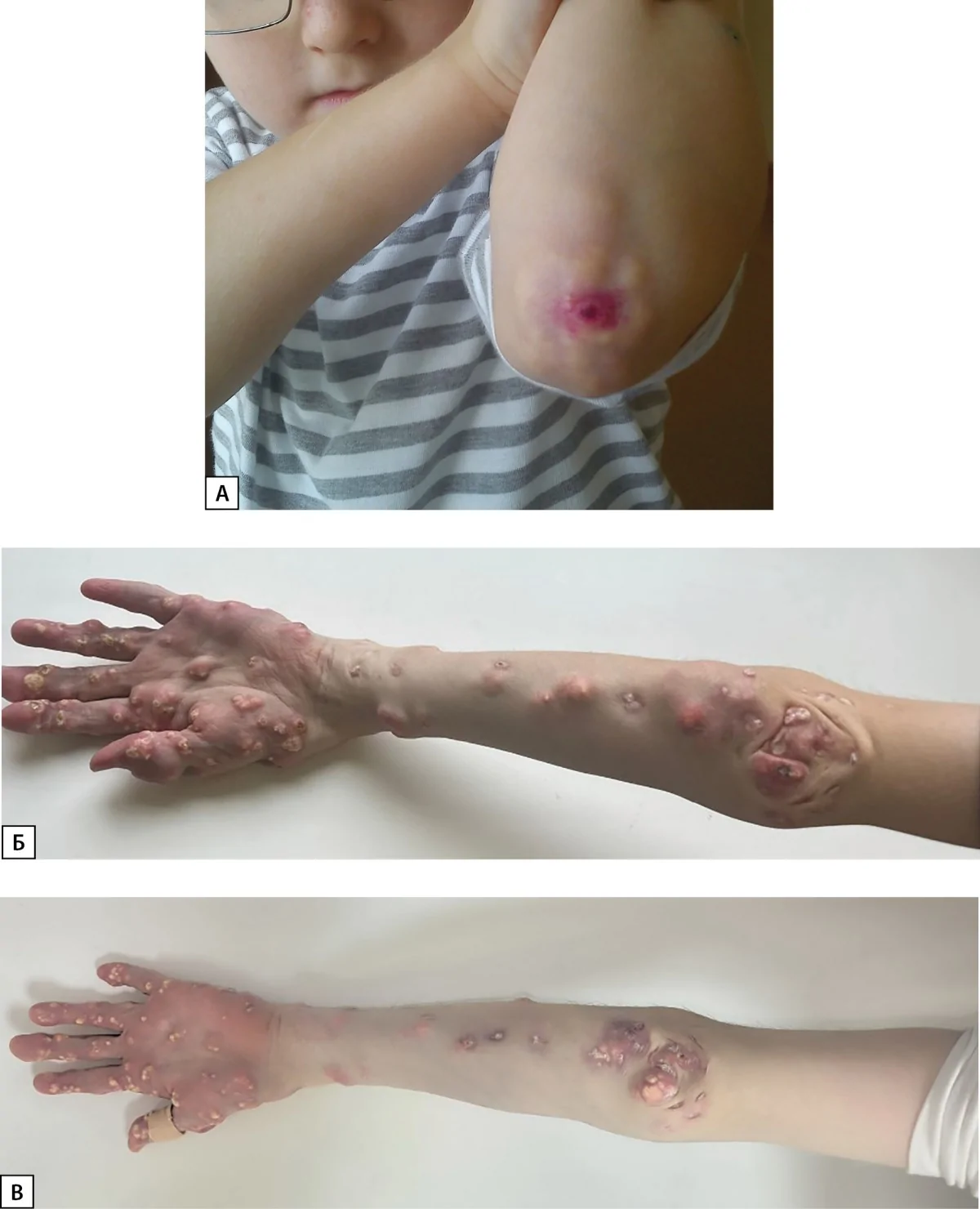
Рисунок 1. Кальцинаты на локте в 4 г 10 мес (при первом обращении) (А); на локте, предплечье и ладонной поверхности кисти в 14 лет (до назначения гипофосфатемической терапии) (Б) и в 17 лет (на фоне регулярной гипофосфатемической терапии) (В).

При обследовании исключены эндокринные причины нарушений кальций-фосфорного обмена (табл. 1). Консультация ревматолога и лабораторные данные (ревмопробы, иммунологические маркеры) не подтвердили воспалительного генеза кальциноза.

По данным УЗИ органов брюшной полости, почек, щитовидной железы, сердца и магистральных сосудов значимых патологических изменений, включая кальцинаты, не выявлено. Ранее заподозренный кальцинат аортального клапана не подтвердился. Рентгенография черепа, кистей и пояснично-крестцового отдела позвоночника кальцинатов не выявила. Костный возраст отставал на 1 год (соответствовал 3,5 года), в дальнейшем соответствовал паспортному.

Учитывая прогрессирующий характер кальциноза, обсуждалась возможность терапии бисфосфонатами, однако от их назначения на тот момент решено отказаться.

При последующих госпитализациях с целью поиска кальцинатов внутренних органов обследование проводилось по аналогичному плану.

В 5 лет 7 мес при повторной госпитализации выявлено увеличение количества и объема подкожных кальцинатов, появление их на кистях; показатели лабораторного и инструментального обследования без существенной динамики (табл. 1).

В 7 лет 1 мес отмечено дальнейшее прогрессирование кальциноза кожи — кальцинаты в области межфаланговых, пястно-фаланговых, лучезапястных суставов, ограничивающие движения в правой кисти; в области локтевых, коленных суставов, дистальных фаланг больших пальцев ног, на задней поверхности шеи, в подбородочной и окологубной области.

В среднем сегменте чашечно-лоханочного аппарата правой почки выявлен кальцинат (табл. 1). По остальным органам и системам — без динамики.

В 8 лет 1 мес при обследовании, по данным УЗИ, сохранялись кальцинаты в правой почке, впервые заподозрен кальцинат в области мошонки справа (табл. 1).

В 9 лет 1 мес продолжился рост количества и размеров подкожных кальцинатов (рис. 2А, 3А, 4А); подтверждено наличие кальцинатов в правой почке и правой половине мошонки (табл. 1). В связи с выявлением конкремента почки рекомендован прием калия-натрия гидроцитрата, однако терапия не проводилась. С данного периода пациент стал периодически обращаться за хирургической помощью в связи с нагноением кальцинатов в области коленных и локтевых суставов.

**Figure:**
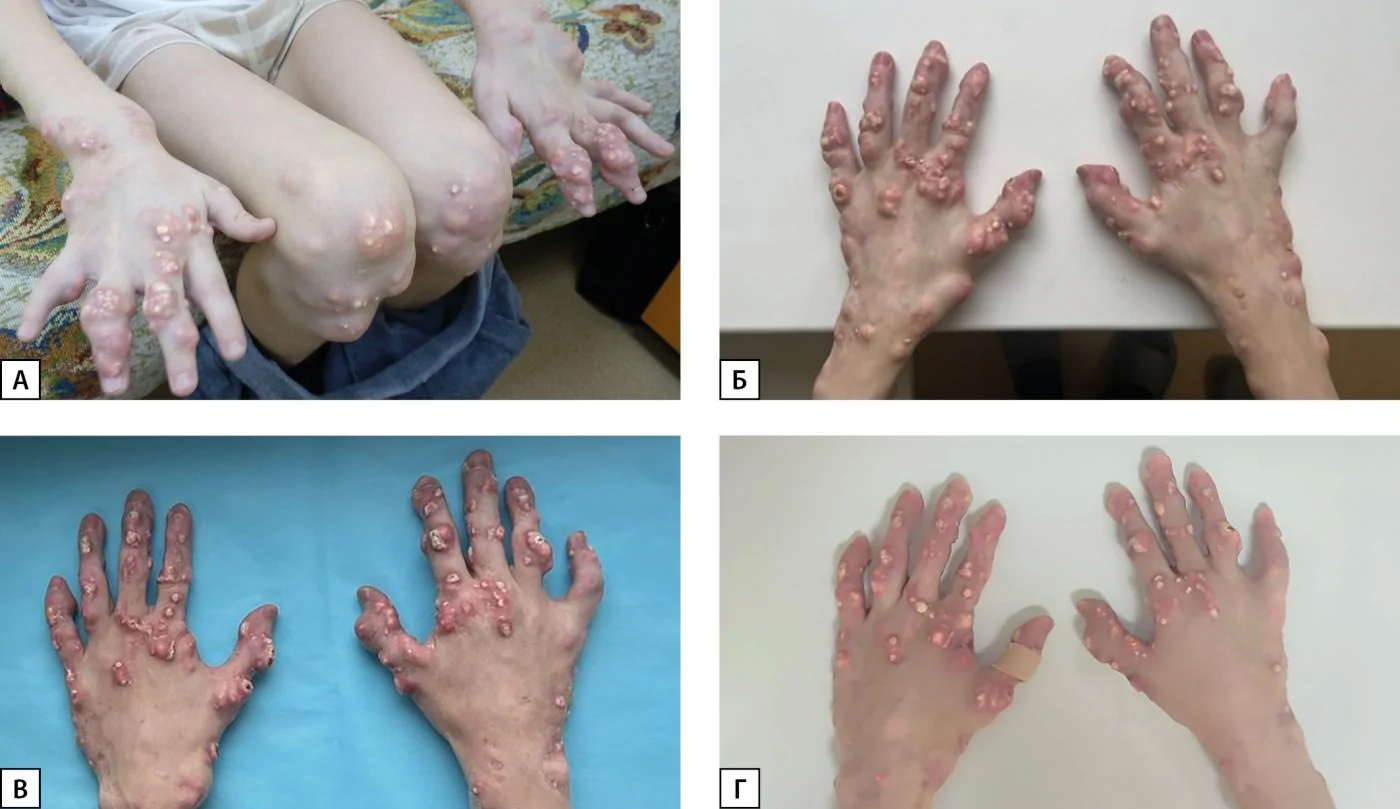
Рисунок 2. Кальцинаты на тыльных поверхностях кистей и коленях в 9 лет (А); на тыльных поверхностях кистей до назначения гипофосфатемической терапии в 14 лет (Б), на фоне нерегулярного приема гипофосфатемических препаратов в 16 лет (В) и в 17 лет на фоне регулярной гипофосфатемической терапии (Г).

**Figure:**
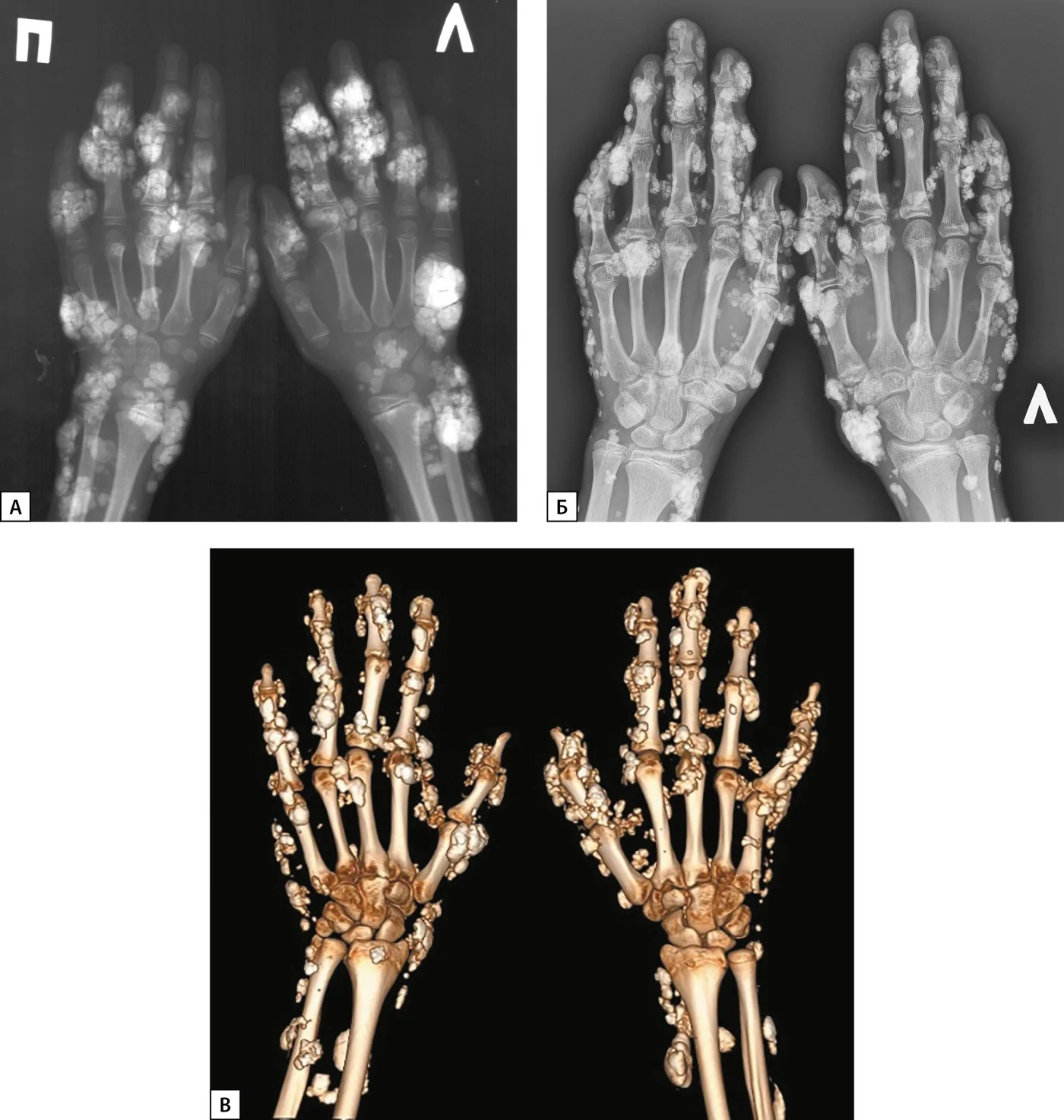
Рисунок 3. Рентгенограммы кистей в 9 лет (А) и 16 лет (Б); компьютерная томограмма кистей в 17 лет (В).

**Figure:**
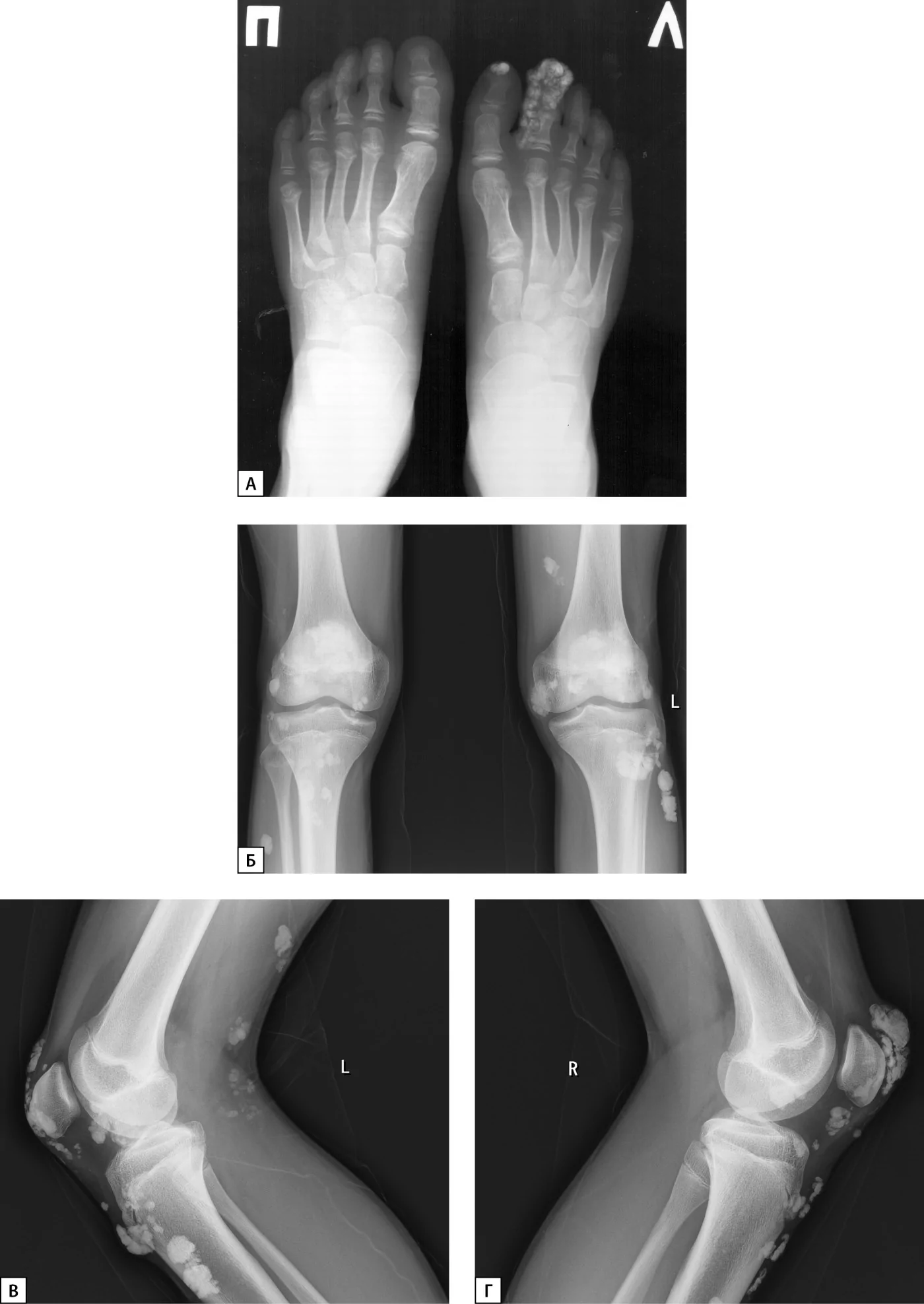
Рисунок 4. Рентгенограммы стоп в 9 лет (А), коленей в 16 лет (Б–Г).

В 10 лет 1 мес при осмотре, помимо множества кальцинатов, отмечено наличие язвенных дефектов кожи в области правого локтевого сустава; по данным УЗИ сохранялись кальцинаты в правой почке и в области мошонки справа (табл. 1).

В 11 лет 2 мес выявлены подкожные кальцинаты в правой ягодичной области, язвенные дефекты после их частичного самопроизвольного вскрытия. При УЗИ не обнаружен наблюдавшийся ранее конкремент правой почки; в области мошонки кальцинат сохраняется (табл. 1).

В 12 лет 5 мес в период госпитализации, помимо ежегодно повторявшегося ультразвукового и рентгенологического обследований с целью поиска кальцинатов, проведены МРТ головного мозга и области гипофиза, а также гормональное исследование — патологических изменений не выявлено (ИФР — 1 127 нг/мл (N 95–460), пролактин — 271 мкМЕ/мл (N 45–375), ТТГ — 2,3 мкМЕ/мл (N 0,5–6,0), свТ4 — 17,3, пмоль/л (N 10,3–24,5)). По данным УЗИ, кальцинат в мошонке — без существенной динамики. После выявления выраженного дефицита 25-ОН-витамина Д (табл. 1) была рекомендована терапия препаратами нативного витамина Д в дозе 1000 МЕ, которую пациент периодически получал в течение следующих двух лет.

В 13 лет 3 мес отмечено начало полового созревания — при осмотре соответствует стадии Таннер 2, тестикулы по 10 мл. Отмечено увеличение числа кальцинатов на лице (лоб, щеки, подбородок, ушные раковины) — в местах, типичных для юношеского акне. По данным УЗИ, в мошонке обнаружен еще один кальцинат (табл. 1).

В 14 лет 3 мес кальцинаты сохранялись в прежнем количестве (рис. 1Б, 2Б); отмечено прогрессирование полового созревания (Таннер 3, тестикулы 12–15 мл). В области мошонки сохраняются два кальцината. Проведена денситометрия поясничного отдела позвоночника — выявлена выраженная остеопения (Z-score -2,4), рекомендовано продолжить прием препаратов витамина Д в постоянном режиме под контролем показателя 25-ОН-витамина Д.

## Результаты молекулярно-генетического исследования

В возрасте 6 лет проведено молекулярно-генетическое исследование (панель Ampliseq_Rickets — гены FGF23, PTHR1, CASR, SLC34A1, ATP6V1B1, SLC9A3R1, SLC34A3, CYP24A1, ATP6V0A4, GALNT3, ALPL, PHEX, CYP27B1, LRP5, DMP1, VDR, KL, CYP2R1, ENPP1, CLCN5, CLCNKB, SLC2A2, общее покрытие 98,5%) — патологически значимых нарушений нуклеотидной последовательности не выявлено.

По данным секвенирования экзома пациента (в возрасте 14 лет), выявлена гомозиготная мутация p.E1200K в гене SAMD9 — вариант неопределенной клинической значимости (мутации гена SAMD9 описаны при нормофосфатемическом семейном опухолевом кальцинозе); в гене GNAS в интроне 11 выявлен гетерозиготный вариант c.971-54C>A (доминантные мутации гена GNAS описаны при прогрессирующей гетеротопической оссификации).

У матери и отца пациента при секвенировании экзома в гене SAMD9 (NM 017654.4) в экзоне 3 выявлен гетерозиготный вариант c.3598G>A: p.E1200K. У матери пациента в гене GNAS (NM 000516.7) в интроне 11 выявлен гетерозиготный вариант c.971-54C>A.

## Дифференциальный диагноз

Проведенное клинико-лабораторное обследование в течение всего периода наблюдения позволило исключить наиболее распространенные причины кальциноза — нарушения кальций-фосфорного обмена и вторичный кальциноз кожи на фоне хронических воспалительных заболеваний. До получения результатов молекулярно-генетического исследования диагноз трактовался как идиопатический кальциноз кожи, а после — как нормофосфатемический семейный опухолевый кальциноз.

## Лечение

В 14 лет 3 мес с целью снижения уровня фосфора пациенту был рекомендован прием алюминия гидроксида, на фоне терапии которым после выписки из стационара отмечено снижение фосфора до 1,2 ммоль/л, а в дальнейшем — вновь повышение до 1,8 ммоль/л (табл. 1).

В 15 лет 4 мес на фоне дальнейшего прогрессирования полового созревания (Таннер 4) сохраняется большое количество кальцинатов, в том числе на лице — в областях, типичных для юношеского акне. При проведении эхокардиографического исследования выявлено неравномерное уплотнение клапана легочной артерии, не позволяющее исключить наличие кальцината.

Несмотря на рекомендованный прием алюминия гидроксида, при поступлении в стационар выявлен повышенный уровень фосфора 1,74 ммоль/л, что объясняется нерегулярным приемом препарата. С целью снижения уровня фосфора врачебной комиссией было принято решение о терапии препаратом гипофосфатемического действия севеламер в начальной дозе 400 мг 2 раза в сутки. В период пребывания в стационаре под контролем показателей кальций-фосфорного обмена проводилось постепенное увеличение дозы севеламера до 800 мг * 3 раза в сутки. Терапия севеламером прекращена пациентом самостоятельно через 2 месяца после выписки из стационара в связи с жалобами на мышечную слабость.

В 16 лет все ранее выявленные кальцинаты сохраняются (рис. 2В, 3Б, 4Б–Г). Несмотря на нерегулярный прием препаратов гипофосфатемического действия, отмечено визуальное уменьшение размеров кальцинатов на кистях и размера кальцината в мошонке по данным УЗИ (табл. 1). С учетом жалоб пациента решено комбинировать прием гидроксида алюминия утром и днем (по 1 саше) с вечерним приемом севеламера 800 мг.

## Исход и результаты последующего наблюдения

В 17 лет зафиксировано значительное визуальное уменьшение количества и размеров кальцинатов (рис. 1В, 2Г, 3В). В течение года не отмечалось эпизодов гнойного воспаления кальцинатов. Пациент связал улучшение с регулярным приемом терапии. При обследовании отмечено снижение уровня фосфора; сохранялись кальцинаты в мошонке; эхокардиографические признаки возможных микрокальцинатов в структурах сердца (табл. 1). С целью дальнейшего подавления образования кальцинатов рекомендован прием севеламера в дозе до 3200 мг/сут под контролем показателей кальций-фосфорного обмена.

## ОБСУЖДЕНИЕ

## Этиология опухолевого кальциноза кожи

Первое описание семейного опухолевого кальциноза под названием «кальцифицированный эндотелий» (endotheliome calcifié) относится к 1898 г. и принадлежит французским дерматологам Giard M.H. и Duret A. Позднее, в 1935 г., немецкий дерматолог Tseutschlaender O. проанализировал серию клинических случаев этого заболевания и назвал его «липокальциногрануломатоз» (lipocalcinogranulomatosis). В 1943 г. Inclan A. с соавт. описали кальциноз в американской литературе и отделили это врожденное заболевание от похожих по клиническим проявлениям приобретенных состояний, закрепив за ним название «опухолевый кальциноз» [2].

В 1996 г. Smack D. с соавт. проанализировали описания 121 пациента с кальцинозом, выделив среди них первичный (идиопатический) и вторичный (на фоне заболевания, которое могло спровоцировать отложение солей кальция). В зависимости от уровня фосфора в крови первичный кальциноз был разделен на два заболевания — гиперфосфатемический (ГСОК) и нормофосфатемический семейный кальциноз (НСОК). Было продемонстрировано, что для НСОК характерна манифестация в первой декаде жизни (манифестация ГСОК возможна во второй декаде), большинство пациентов проживало в тропиках или субтропиках (пациенты с ГСОК были преимущественно африканского происхождения), обычно отсутствовал семейный анамнез заболевания [3].

ГСОК ассоциирован с мутациями в трех генах: FGF23 (фактор роста фибробластов 23, регулирует выведение фосфора с мочой), KL (является корецептором для FGF23) и GALNT3 (кодирует гликозилтрансферазу, ответственную за гликозилирование FGF23). Патология одного из них может приводить к гиперфосфатемии и эктопической кальцификации [2][4].

НСОК характеризуется отсутствием метаболических нарушений и ассоциирован с мутациями в гене SAMD9. Его распространенность значительно ниже, чем ГСОК. Описаны единичные семьи пациентов с НСОК среди йеменских евреев и ряд спорадических случаев [5–7].

В 2006 г. Topaz O. с соавт. при изучении 8 пациентов с НСОК из 5 еврейских семей йеменского происхождения выделили гомозиготный интервал в области 7q21-q21.3. В гене SAMD9, расположенном на этом участке, у пациентов с НСОК был выявлен гомозиготный вариант K1495E. Кроме этого, при обследовании 92 здоровых лиц в еврейских семьях, иммигрировавших в Израиль из Йемена, был выявлен 1 носитель данного патогенного варианта в гетерозиготном состоянии [5].

В 2008 г. Chefetz I. и соавт. описали двух дизиготных близнецов с НСОК, обусловленным компаунд-гетерозиготной мутацией SAMD9 (кроме ранее описанного патогенного варианта K1495E, был выявлен R344X) [6].

SAMD9 (sterile alpha motif domain 9) — это цитоплазматический белок, состоящий из 1589 аминокислот, экспрессирующийся во всех тканях, кроме мозга и мускулатуры плода. Его активность снижена в целом ряде опухолей и ассоциируется с усилением клеточной пролиферации и подавлением апоптоза. Предполагается, что SAMD9 участвует в регуляции воспаления в коже, в частности контролирует апоптоз, индуцированный фактором некроза опухоли альфа (ФНО-α) [6].

Мутации в SAMD9 были описаны также при ряде онкологических заболеваний и первичных иммунодефицитных состояниях. В частности, они приводят к развитию MIRAGE-синдрома (Myelodysplasia (миелодисплазия), Iinfections (инфекции), Restriction of growth (дефект роста), Аdrenal hypoplasia (гипоплазия надпочечников), Genital phenotypes (нарушение формирования пола), Еnteropathy (энтеропатия)) [8][9].

Нашему пациенту выполнено секвенирование экзома, по результатам которого выявлен гомозиготный вариант p.E1200K в SAMD9, тогда как у обоих родителей этот вариант обнаружен в гетерозиготном состоянии. Такая конфигурация соответствует аутосомнорецессивному типу наследования, характерному для SAMD9-ассоциированных форм НСОК, однако конкретный вариант p.E1200K в доступных базах и публикациях не описан как патогенный и в настоящее время классифицируется как вариант неопределенного клинического значения (VUS). Учитывая выраженную клиническую картину с множественными кальцинатами и ранним началом заболевания, совокупность данных позволяет рассматривать выявленный вариант SAMD9 как вероятную генетическую основу заболевания, хотя окончательная валидация его патогенности требует накопления дополнительных клинических и функциональных данных.

Помимо этого, у пациента был идентифицирован гетерозиготный интронный вариант c.97154C>A в гене GNAS, обнаруженный также у матери. Патогенные GNASмутации при прогрессирующей гетеротопической оссификации и других GNAS-ассоциированных синдромах обычно затрагивают кодирующие участки или канонические сплайссайты и приводят к инактивирующим изменениям Gsα. Описаний варианта c.97154C>A как патогенного в литературе нет, а отсутствие типичных GNAS-ассоциированных фенотипических проявлений у матери позволяет расценивать данный вариант как VUS с вероятно низкой клинической значимостью в контексте представленного случая.

## Лечение опухолевого кальциноза кожи

НСОК обычно клинически манифестирует в первые годы жизни. Появлению кальцинатов предшествует развитие воспалительных элементов (обычно вследствие травматизации), на месте которых быстро образуются кальцифицированные узелки, которые могут изъязвляться, приводя к эвакуации содержимого, внешне напоминающего мел [10].

К наиболее типичным местам локализации кальцинатов относят верхнюю конечность (плечо, локоть) и бедро. Также они могут располагаться в области спинальных отростков, вокруг ниже-челюстного сустава и мелких суставов кистей и стоп, а также в подколенной ямке [2].

Предполагается, что в патогенезе образования кальцинатов ведущую роль играет хроническая травматизация и транзиторная гиперфосфатемия [4].

Терапия НСОК в большинстве случаев рассматривается с учетом опыта лечения более распространенных вариантов заболевания — вторичного кальциноза кожи при ревматических заболеваниях и ГСОК. Хирургическое лечение позволяет избавляться от кальцинатов, но не предотвращает рецидивы и может приводить к таким осложнениям, как позднее заживление, формирование свищей и карманов, вторичное инфицирование [10][11]. В качестве альтернативного метода предлагается микроигольчатая терапия [12]. Существует ряд публикаций, демонстрирующих уменьшение вторичного кальциноза кожи при местном применении препаратов натуральных масел [13], тиосульфата натрия [14][15] и пиросульфита натрия [11] в сочетании с хирургическим удалением и экстракорпоральной ударно-волновой литотрипсией [16].

При вторичном кальцинозе кожи также обсуждается назначение различных противовоспалительных препаратов, блокаторов кальциевых каналов, колхицина, варфарина, ритуксимаба, тофацитиниба [1][17]. Было продемонстрировано, что примерно у половины пациентов с вторичным кальцинозом на фоне ревматических заболеваний эффективно назначение бисфосфонатов [1][18].

Для лечения ГСОК в качестве наиболее перспективных обсуждаются терапевтические методы снижения уровня фосфора в крови. С этой целью возможно ограничение потребления фосфора с пищей и применение препаратов, способствующих снижению уровня фосфора — гидроксида алюминия, севеламера, карбоната лантана и ацеталоамида. Существует мнение, что такое лечение патогенетически обосновано не только при ГСОК, но и при НСОК [4].

В 2021 г. был опубликован клинический случай с положительным опытом применения севеламера в дозе 400 мг в течение 6 месяцев у 14-летнего пациента с множественными кальцинатами в области подмышечной впадины, колена и шеи [19].

Особенностью нашего пациента является наличие множественных кальцинатов не только в местах травматизации (конечности), но и в местах угревой сыпи (лицо), а также особенно активное прогрессирование заболевания в период полового созревания.

Стараясь не провоцировать образование новых кальцинатов, мы длительное время избегали назначения препаратов витамина Д. На фоне дефицита витамина Д и постоянной потери кальция и фосфора у нашего пациента выявлена выраженная остеопения, хотя в описании клинических случаев НСОК не сообщается о развитии остеопороза у пациентов [5][6], что требует дальнейшего наблюдения.

Предложенная в публикации доза севеламера 400 мг в сутки [19] не позволила нашему пациенту нормализовать уровень фосфора. Для его снижения в условиях стационара требовались стандартные дозы, обычно рекомендуемые взрослым пациентам при гиперфосфатемии 1,76–2,42 ммоль/л — по 800 мг 3 раза/сут [20].

Учитывая уменьшение размеров кальцината мошонки по данным УЗИ и визуальное уменьшение кальцинатов на кистях, можно сделать вывод, что даже небольшое снижение концентрации фосфора в крови позволяет уменьшить образование новых кальцинатов.

Эффективность проводимой терапии дискутабельна, ограничена как плохой переносимостью севеламера пациентом, так и недостаточной приверженностью к лечению в течение первого года терапии.

Целью дальнейшего наблюдения данного пациента является подбор терапии, которая бы позволила, не повышая риска остеопороза, удерживать уровень фосфора в рамках референсных значений и уменьшить количество и размеры кальцинатов.

## ЗАКЛЮЧЕНИЕ

Описанный клинический случай демонстрирует пациента с чрезвычайно редким заболеванием, нормофосфатемическим семейным опухолевым кальцинозом, обусловленным мутациями гена SAMD9. Это первое в России описание пациента с данным диагнозом, подтвержденным молекулярно-генетически. Точная постановка диагноза позволила выявить патогенетический механизм образования кальцинатов и предложить варианты терапии.

## ДОПОЛНИТЕЛЬНАЯ ИНФОРМАЦИЯ

Источники финансирования. Работа выполнена по инициативе авторов без привлечения финансирования

Конфликт интересов. Авторы декларируют отсутствие явных ипотенциальных конфликтов интересов, связанных с содержанием настоящей статьи.

Участие авторов. Все авторы одобрили финальную версию статьи перед публикацией, выразили согласие нести ответственность за все аспекты работы, подразумевающую надлежащее изучение и решение вопросов, связанных с точностью или добросовестностью любой части работы.

Согласие пациента. Пациент и его родитель добровольно подписали информированное согласие на публикацию персональной медицинской информации в обезличенной форме в журнале «Проблемы эндокринологии».
